# The Usability of the Preliminary ICF Core Set for Hospitalized Patients After a Hematopoietic Stem Cell Transplantation From the Perspective of Nurses: A Feasibility Study

**DOI:** 10.3389/fresc.2021.710127

**Published:** 2021-08-13

**Authors:** Hillegonda A. Stallinga, Janita Bakker, Sylvia J. Haan, Harmieke van Os-Medendorp, Marijke C. Kars, Louis Overgoor, Roy E. Stewart, Petrie F. Roodbol

**Affiliations:** ^1^Department of Health Sciences, Nursing Science, University Medical Center Groningen, University of Groningen, Groningen, Netherlands; ^2^Department of Oncology, Isala Clinic, Zwolle, Netherlands; ^3^Department of Hematology, University Medical Center Groningen, Groningen, Netherlands; ^4^School of Health, Saxion University of Applied Sciences, Deventer/Enschede, Netherlands; ^5^Nursing Science, Program in Clinical Health Sciences, University Medical Center Utrecht, Utrecht University, Utrecht, Netherlands; ^6^Bettery Institute, Diemen, Netherlands; ^7^Department of Public Health, University Medical Center Groningen, University of Groningen, Groningen, Netherlands

**Keywords:** ICF core set, hematology, stem cell transplantation, feasibility, functioning

## Abstract

**Background:** A hematopoietic stem cell transplantation (HSCT) has a major impact on the functioning and perceived quality of life of patients. To describe the functioning of patients, a preliminary set of 53 categories of the International Classification of Functioning, Disability and Health (ICF) as relevant for HSCT patients has been selected earlier by a Delphi study. For the implementation of this preliminary ICF core set for patients after HSCT in clinical practice, a feasibility study was requested.

**Methods:** A feasibility study was conducted in an explanatory mixed-methods research design. Qualitative data were collected cross-sectionally by semi-structured interviews based on specific topics related to feasibility regarding the use of the preliminary ICF core set for HSCT patients from the perspective of nurses (five in ICF-trained nurses and five regular, untrained, nurses). Quantitative data, were collected longitudinally by using a mobile health application based on ICF in which the ICF trained nurses registered HSCT patients' functioning.

**Results:** Qualitative analysis indicated that using the preliminary ICF core set is practical and acceptable for providing information about the functioning of HSCT patients from the perspective of nurses. In addition, nurses indicated a demand for this information due to its impact on multidisciplinary meetings and clinical decision-making by involving relevant aspects of the functioning of patients. Management support, trained staff, and designated time to focus on functioning are mentioned as requirements for successful implementation. Quantitative analysis demonstrated that the most used 30% (*n* = 17) ICF categories are included in the preliminary ICF core set for HSCT patients (*n* = 24). Energy (b130) was the most used ICF category. Family relationships (d760) was the most frequently and highly positively associated ICF category.

**Conclusions:** From the perspective of nurses, the preliminary ICF core set for HSCT patients is feasible and relevant in gaining information regarding functioning. Applying this preliminary ICF core set for HSCT patients in the anamnesis and the nursing consultations contributes to this information. Further research is needed to look at the perspective of other professionals and HSCT patients themselves.

## Introduction

More than 50,000 hematopoietic stem cell transplantations (HSCTs) per year are reported worldwide, and ~20,000 of these take place in Europe (1). Stem cells can be harvested from the patient (autologous) or from a matched donor (allogeneic) (2) and are dosed by an intravenous infusion (3). Stem cell transplantation is a treatment option for hematological malignancies as well as a number of other malignant and non-malignant diseases (1). The side effects of stem cell transplantation are medical symptoms like infections and organ dysfunction (4). Furthermore, there are side effects with an impact on the perceived quality of life, such as fatigue, anxiety, depressive symptoms, and sexual dysfunction (5, 6).

The primary purpose of HSCT from a medical perspective is to improve the health of an individual by curing the disease. However, the health of a patient after a HSCT also includes his or her experienced physical and psychosocial functioning (4). Therefore, healthcare provision of patients after a HSCT should be focused on functioning as well (7). The International Classification of Functioning, Disability and Health (ICF) is a classification that can be used as a tool to focus on functioning and supporting self-management of patients (8).

**Figure 1:**
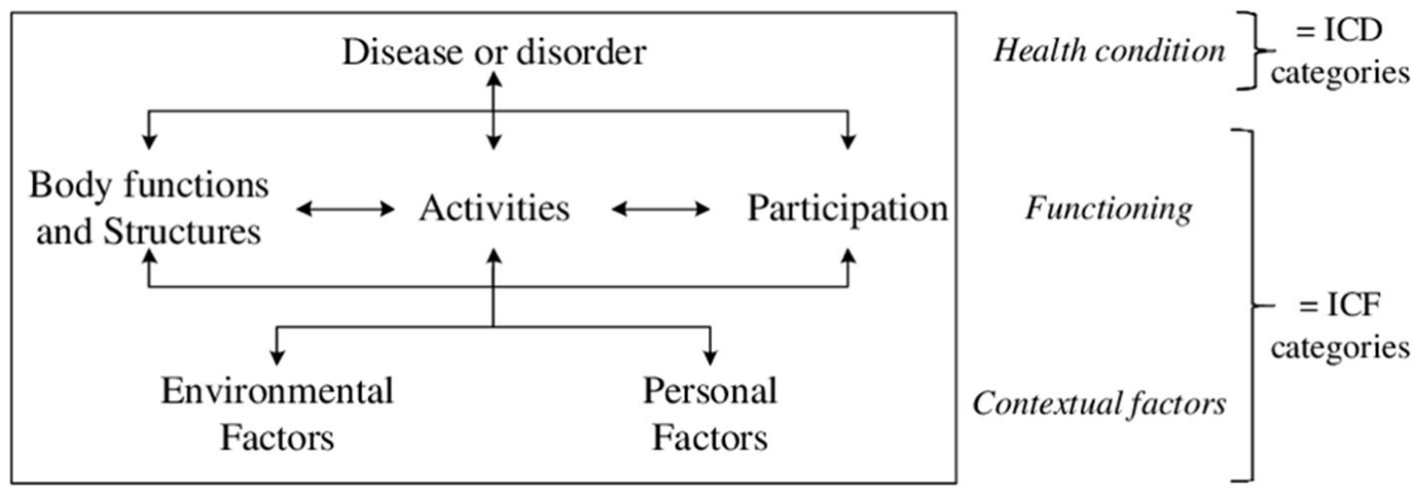
WHO's conceptual model of health representing the interactions between the components (disease, body functions and structures, activities, participation, environmental and personal factors) of the health status. ICD, International Classification of Diseases; ICF, International Classification of Functioning, Disability and Health (8).

The World Health Organization (WHO) published the conceptual model of health as presented in Figure 1, in which the ICF and the International Statistical Classification of Diseases and Related Health problems (ICD) are included to describe the health of an individual (9). Functioning is conceptualized as a result of the dynamic interaction between a health condition (disease or disorder) and contextual factors (environmental and personal factors) (9). Functioning pertains to the functions of people, the performance of activities, and the areas of life in which they participate. The ICD classifies health conditions, whereas the ICF classifies functioning and environmental factors influencing functioning. Personal factors are lacking in the current ICF but, although different articles are published with preliminary lists (10), the WHO has decided to refrain from a classification of personal factors in the near future. The ICF is complementary to the ICD; both classifications are essential in completing the description of health (9).

The WHO's conceptual model of health is based on a biopsychosocial model. Using this model broadens the focus of healthcare from disease (biomedical) to functioning and contextual factors (biopsychosocial) (9, 11, 12). The biopsychosocial perspective fits the holistic orientation (8, 13), which is requested for person-centered care as a central theme in nursing and allied health professionals. Moreover, using the ICF will facilitate all professionals and disciplines to be effective partners in multidisciplinary teams due to the standard terminology of ICF (14). Implementing the ICF may be a first step in delivering healthcare with a biopsychosocial approach in which functioning is the focus of health care (8). Limitations related to implementing the ICF are demonstrated in relation to the utility of the ICF for routine practice (15). Tools that are simple to use are mentioned as an important step in this process (16, 17).

The ICF includes more than 1,500 categories. Each of these categories describes an aspect of functioning, for example, work and sleep. Every ICF category has a code, such as b134 (sleep) or d845 (work). These codes are referring to the categories of the ICF. To make the ICF feasible in daily practice, core sets are developed (18, 19). Core sets are sets of selected ICF categories related to a specific health condition (20, 21). For example, there are core sets for hearing loss (22), head and neck cancer (23), and stroke (24). Due to the lack of an ICF core set for HSCT patients, a development project has begun in 2015 in the Netherlands.

Following the guideline for the development of an ICF core set (20), a content analysis, a literature review, and a Delphi study were conducted (25). Currently, the first version, the preliminary ICF core set for HSCT patients (Table 1), needs to be explored and tested to examine the relevance in clinical practice. A feasibility study from the perspective of nurses was conducted, since nurses are the primary healthcare professionals involved in the functioning of patients (26).

**Table 1:** Preliminary ICF core set for HSCT patients.

**Component**	**Chapter**	**Categories**	**Code**
Body Functions	1. Mental functions	1. Energy/sleep/attention/memory/emotion/thought	1. b130/b134/b140/b144/b152/b160
	2. Sensory functions and pain	2. Taste/sensation of pain	2. b250/b280
	4. Functions of the cardiovascular, hematological, immunological, and respiratory systems	4. Heart functions/blood pressure/hematological system/immunological system/exercise tolerance	4. b410/b420/b430/b435/b455
	5. Functions of the digestive, metabolic, and endocrine systems	5. Ingestion/defecation/weight maintenance/ sensations associated with the digestive/ water, mineral, and electrolyte balance/thermoregulatory/ endocrine gland functions	5. b510/b525/b530/b535/b545/b550/b555
	6. Genitourinary and reproductive functions	6. Sexual functions	6. b640
	7. Neuromusculoskeletal and movement-related functions	7. Muscle power/sensations related to muscles and movement	7. b730/b780
	8. Functions of the skin and related structures	8. Protective functions/repair functions/hair/nails	8. b810/b820/b850/b860
Activities and Participation	2. General tasks and demands	2. Undertaking a single task/ handling stress and other psychological demands	2. d210/d240
	5. Self-care	5. Washing/ caring for body parts/ dressing/ looking after health of an individual	5. d510/d520/d540/d570
	6. Domestic life	6. Preparing meals/doing housework/caring for household objects	6. d630/d640/d650
	7. Interpersonal interactions and relationships	7. Basic interpersonal interactions/complex interpersonal interactions/ informal social relationships/family/intimate relationships	7. d710/d720/d750/d760/d770
	8. Major life areas	8. Job employment/remunerative employment/economic self-sufficiency	8. d845/d855/d870
	9. Community, social, and civic life	9. Community life/recreation and leisure	9. d910/d920
Environmental Factors	3. Support and relationships	3. Immediate family/friends/ personal care providers and personal assistants/health professionals	3. e310/e320/e340/e355
	5. Services, systems, and policies	5. Legal services, systems, and policies/social security, services, systems, and policies/health services, systems, and policies	5. e555/e570/e580

The aim of this study was to evaluate the preliminary ICF core set for HSCT patients on its feasibility and to perform a quantitative exploration related to the use of ICF categories by nurses in the care of hematological patients hospitalized for autologous and allogeneic stem cell transplantation.

## Methods

### Design

A feasibility study was performed to determine the usability of the preliminary ICF core set for HSCT patients (27). A qualitative-dominant, explanatory mixed-methods design was used to gain an insight into the usability with both quantitative and qualitative data (28). First, longitudinal quantitative data were collected by using the preliminary ICF core set for HSCT patients. Second, qualitative data were collected on specific topics related to the feasibility and the use of the preliminary ICF core set for HSCT patients. Qualitative data of the experience logs of nurses were collected to minimize the recall bias (28).

### Population and Study Procedure

The study population consists of ICF-trained nurses and regular nurses working with patients hospitalized for HSCT at the Department of Hematology in a University Medical Centre in the northern parts of the Netherlands. Registered Dutch-speaking nurses with a minimum of 1 year of hematological experience were included. To be effective due to the limited number of ICF-trained nurses at the department who can serve as primary data sources, a purposeful sample was used. All the five ICF-trained nurses were included as a homogenous group supplemented with five regular nurses to ensure a collection of data with different perspectives regarding the usability of the preliminary ICF core set. Nurses were not eligible if they took care of fewer than two of the included patients during the test period. A convenient sample of all inpatients hospitalized for stem cell transplantation on January 16, and March 31, 2017, was included in the study (28).

**Figure 2:**
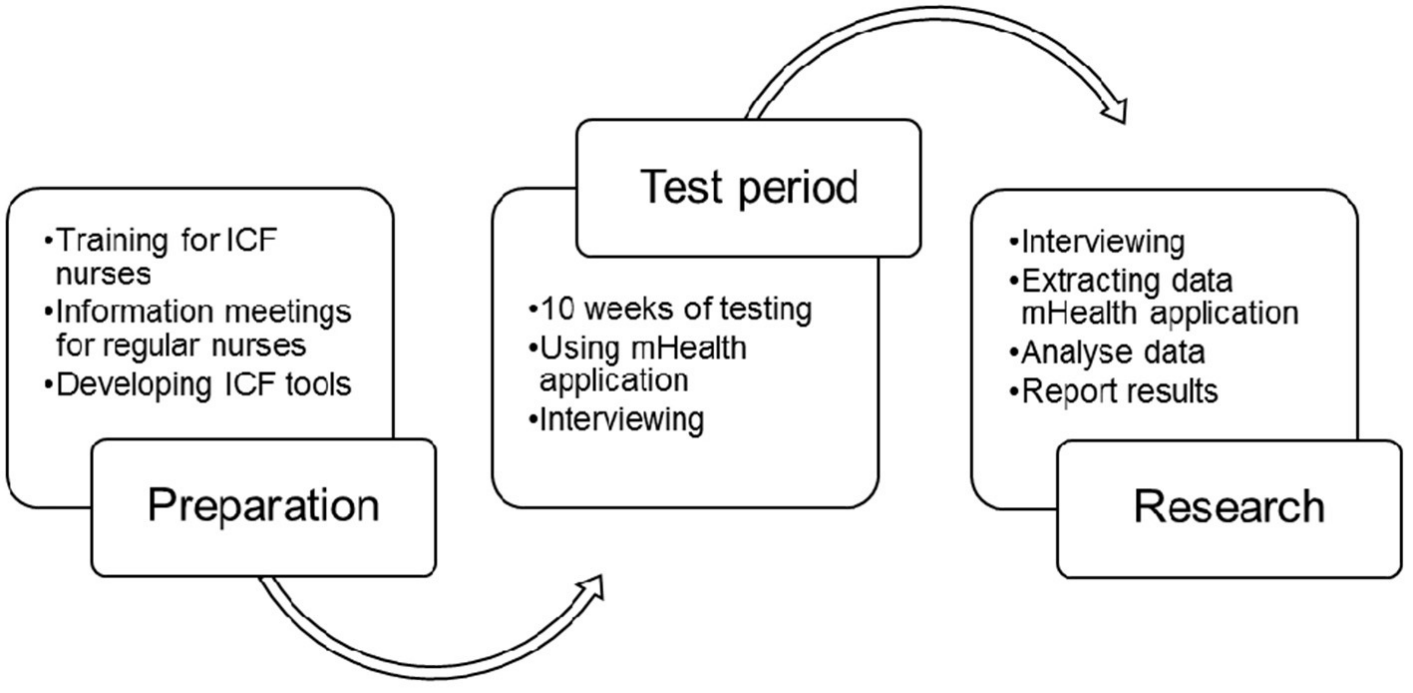
Timeline research procedure. Both the preparation and research phase lasted 8 weeks.

In the study procedure (see Figure 2 for the timeline research procedure), the five ICF nurses, selected by the head nurse of the hematology department as “ICF nurse,” were trained by the ICF expert (HS) in using the preliminary ICF core set for HSCT patients, in doing the nursing consultations and in registration patients' functioning in the mobile health (mHealth) application. The training consisted of an every 3-week 4-h instructor-led discussion-based training in using the ICF. This training is part of an existing ICF training course developed and given over the past several years in the Netherlands (29). The format of the training is based on the concept of meaning and application-oriented learning, indicating that the training appeals to the experience of healthcare professionals and focuses on the practical application of the ICF in patient care. The regular nurses [not trained in ICF, but in daily practice confronted and as such familiar with ICF registrations in patient files and (multi)disciplinary consultations] of the department received information about the ICF project in departmental staff meetings. A new patient nursing anamnesis tool, based on the preliminary ICF core set for HSCT patients, was developed. By using the mHealth application, which includes the entire ICF including the preliminary ICF core set for HSCT patients, ICF nurses register the functioning of patients during the admission of patients and nursing consultations (twice a week on average) in the relevant ICF categories. In clinical practice, this means that the admission is started and registered from the preliminary core set and ends with the open-ended question of whether anything relevant to the functioning of patients should be added. The consultations are exactly the other way around: They start open and the patient can come up with anything that is relevant with regard to functioning. This is registered, and depending on previous consultations, both the nurse and the patient can return to and also use the categories of the preliminary core set. Regular nurses did not use the mHealth application, nor did they perform patient admissions or nursing consultations.

The test phase lasted for a period of 10 weeks in which the preliminary ICF core set for HSCT patients was used in clinical practice. Reports of the patient nursing anamnesis, the nursing consultations, and the experience of the ICF nurses with the use of the ICF core set were included in the logs of the mHealth application. A report of the patient nursing anamnesis and the results of the nursing consultations were added to the patient record to inform the regular nurses.

After 5 weeks of the test period, the first data and experiences regarding the use of the preliminary ICF core set were extracted from the mHealth application. The use of the ICF categories and the logged experiences were analyzed to complete the interview guide. A pilot interview was conducted to enhance the topics of the interview guide. The following topics were identified: practicality (structure, time investment, usability), implementation (design, facilitators, barriers), acceptance (positive, negative), demand (use of the ICF core set), and use of ICF categories (frequency, relevance, usability). The interviews were audiotaped and transcribed. The transcripts of the interviews were matched with the audiotapes. A written member check was performed after the qualitative analysis to determine the authenticity of the results (28). The qualitative analysis was matched with the experiences logs of the nurses to prevent recall bias. The remaining quantitative and qualitative data of the mHealth application were extracted at the end of the test period. For the interviews, the five ICF nurses were included, and also five regular nurses were recruited by email.

### Data Collection

The quantitative data related to the use of the ICF categories were collected with the mHealth application (30). The data were extracted twice: at the mid and the end of the test period. Data were collected on the frequency of the use of ICF categories and the qualifier (negative, normal, and positive). These qualifiers in the mHealth application were assigned by the ICF nurses in consultation with the patient.

The qualitative data were collected by a mHealth application (30) and by interviews. ICF nurses reported their experiences with using the nursing anamnesis form and the nursing consultations in the log of the mHealth application (30). Semi-structured interviews with ICF nurses and regular nurses were conducted by researcher JB. The interviews were based on an interview guide and were performed in April 2017. The interview guide contained four focus areas preferred for this feasibility study: acceptability, practicality, implementation, and demand (31). The quantitative data regarding the use of ICF categories (frequency, relevance, usability) including the preliminary ICF core set for HSCT patients were added to the interview guide (fifth topic) to ensure the discussion of the use of the preliminary ICF core set for HSCT patients.

### Data Analysis

The quantitative and qualitative data were analyzed separately. Quantitative data were analyzed using the software SPSS24 (IBM Corporate, New York, United States) and SAS (SAS Institute, North Carolina, United States). The ICF categories were analyzed with descriptive statistics by frequency and associations. The associations between the ICF categories used were analyzed with a chi-square test because the association is measured between binary variables (measured with negative–positive qualifiers) in a two-by-two table (32). Effect sizes [phi *(*ϕ*)*] were calculated and assessed against Cohen's criteria (ϕ 0.1 = small, ϕ 0.3 = medium, ϕ 0.5 = large), using Fisher's exact test to determine statistical significance by the *p*-value (32). Pearson's correlation coefficient was used to analyze the association between the 30% most used ICF categories and baseline characteristics such as age and hospital duration. An independent-sample *t*-test was utilized to analyze whether gender and kind of transplantation differ in terms of the top 30% most used ICF categories (32).

Qualitative data were analyzed using the software NVivo11 (QSR International, Melbourne, Australia). Deductive thematic analysis [initial coding (nodes) of the interviews, sorting different nodes into themes, and merging themes into the predefined topics] (33) was applied to the data and enabled the study to report in terms of feasibility by the experiences of the nurses participating in the test period. Intercoder agreement about items and topics was done by the researcher HS and the researcher JB to enhance the reliability of the qualitative analysis.

## Results

Ten nurses, five in ICF-trained nurses (ICF nurses) and five not in ICF-trained nurses (regular nurses), participated in the qualitative part of the study. Table 2 presents the background characteristics of the nurses. No significant differences were found between the background characteristics of the ICF nurses and the regular nurses. A thematic analysis of the interviews resulted in 42 nodes, divided into 10 themes. The themes were linked to the existing topics: use of ICF categories, practicality, demand, acceptability, and implementation.

**Table 2:** Background characteristics of nurses.

	**ICF-trained nurses (*n* = 5)**	**Regular nurses (*n* = 5)**	***P*-value**
Gender (female) *n* (%)	5 (100)	4 (80)	1.000^*^
Age, years, median	38 (28-53)	39 (31-61)	0.421^**^
Work experience years, median	6 (5-35)	17 (6-40)	0.310^**^

* *Fisher's exact test*,

** *Mann–Whitney U-test*.

During the test period, 27 patients were hospitalized. Three patients refused to participate. The quantitative part of the study included 24 patients. Table 3 presents the background characteristics of the patients.

**Table 3:** Background characteristics of patients.

	**Autologous-tx (n = 12)**	**Allogeneic-tx (n = 12)**	***p*-value**
Gender (male) *n* (%)	9 (75)	10 (83)	1.000^*^
Age, years, median (SD)	60 (8)	56 (17)	0.422^**^
Hospital duration, days, mean (SD)	19 (5)	21 (10)	0.573^**^

*Tx, transplantation*

* *Fisher's exact test*,

** *Independent-samples t-test*.

### Use of ICF Categories of the ICF Core Set

In the test period, 100 unique ICF categories are used by nurses for their patients for a total of 1,810 times. Table 4 presents the most used (top 30% = 17 ICF categories) ICF categories. These most used ICF categories, except medication (e110), are included in the preliminary ICF core set for HSCT patients. Out of the most used ICF categories, 11 categories belong to the component of Body Functions and Structures, three to Activities and Participation, and three to Environmental factors. Energy (b130) was the most used ICF category (*n* = 40).

**Table 4:** Frequency of the most used (top 30%) ICF categories.

	**ICF code**	**Description**	**Frequency (Total *n* = 1,810)**	**Percent (%)**
1	b130	Energy	40	2.21
2	b525	Defecation	39	2.15
3	b152	Emotional functions	38	2.10
4	b535	Nausea	37	2.04
5	b280	Pain	35	1.93
6	b430	Hematological system functions	33	1.82
7	b510	Ingestion functions	32	1.77
8	d845	Work	32	1.77
9	e110	Medication	32	1.77
10	b134	Sleep	31	1.71
11	d760	Family relationships	31	1.71
12	e355	Health professionals	30	1.66
13	b455	Exercise tolerance functions	29	1.60
14	b250	Taste	28	1.55
15	d240	Handling stress	28	1.55
16	e310	Immediate family	28	1.55
17	b435	Immunological system functions	27	1.49

Associations between the most used ICF categories are shown in Table 5. Handling stress (d240) is significantly positively associated with family relationships (d760) (ϕ = 0.60, *p* < 0.05), sleep (b134) (ϕ = 0.73, *p* < 0.05), and pain (b280) (ϕ = 0.56, *p* < 0.05). Family relationships (d760) is also significantly positively associated with sleep (b134) (ϕ = 0.63, *p* < 0.05), support of immediate family (e310) (ϕ = 0.60, *p* < 0.05), and immunological system functions (b435) (ϕ = 0.60, *p* < 0.05). Handling stress (d240) and family relationships (d760) are the most frequent significantly associated categories. This means that more problems or strengths in one category are associated with more problems or strengths in another category. The other background characteristics, gender and kind of transplantation, did not differ in terms of the top 30% most used ICF categories.

**Table 5:** Associations [phi *(*ϕ*)* correlation coefficient] between the 30% most used ICF categories ordered by frequency (1 = most used).

**17 immu.fun**.	**b435**																	1.00
**16 immi.fam**	**e310**																1.00	0.24
**15 hand.stres**	**d240**															1.00	0.49^*^	0.24
14 taste	b250														1.00	0.50^*^	0.24	−0.01
13 exerc.toler.	b455													1.00	−0.06	0.18	0.18	0.18
12 health prof.	e355												1.00	−0.37	0.35	−0.10	−0.10	−0.10
**11 fam.rel.**	**d760**											1.00	−0.29	0.26	0.32	0.60^*^	0.60^*^	0.60^*^
**10 sleep**	**b134**										1.00	0.63^*^	−0.06	0.20	0.51^*^	0.73^*^	0.29	0.29
9 medication	e110									1.00	0.19	−0.12	0.45	−0.45	0.24	0.03	−0.19	0.19
8 work	d845								1.00	−0.19	−0.12	−0.19	0.33	−0.34	0.15	−0.06	−0.06	−0.06
7 ingest.func.	b510							1.00	0.06	0.00	0.25	0.19	−0.06	0.20	0.07	0.07	0.07	0.29
6 hemat.func.	b430						1.00	0.49^*^	−0.10	−0.13	0.12	0.08	0.02	0.49^*^	−0.02	−0.02	0.40	0.19
5 pain	b280					1.00	0.07	0.06	−0.17	−0.19	0.41	0.26	−0.22	0.24	0.35	0.56^*^	0.15	0.15
4 nausea	b535				1.00	0.34	0.27	0.12	−0.16	−0.02	0.47^*^	0.19	−0.15	0.34	0.27	0.47^*^	0.27	−0.15
3 emotions	b152			1.00	−0.08	0.25	0.00	0.00	−0.25	0.09	0.35	0.43	0.09	0.00	0.10	0.31	0.10	0.31
2 defecation	b525		1.00	0.08	0.34	0.16	0.07	0.24	−0.17	−0.02	0.59^*^	0.49^*^	−0.22	0.05	0.36	0.36	0.36	0.15
1 energy	b130	1.00	0.54^*^	0.09	−0.02	−0.15	0.31	0.37	0.02	0.07	0.18	0.35	0.12	–.015	0.19	−0.03	0.40	0.19
		b130	b525	b152	b535	b280	b430	b510	d845	e110	b134	**d760**	e355	b455	b250	**d240**	**e310**	**b435**

* *Significantly associated: p <0.05 Fisher's exact test; ϕ, 0.10 small effect, 0.30 medium effect, 0.50 large effect; boldface printed are significantly and highly associated with very large effect. The 30% most used ICF categories, including the full descriptions of the labels, are presented in Table 4*.

In contrast to the quantitative analysis, demonstrating that some categories may be more relevant due to more frequent use than other categories, the qualitative analysis showed that all ICF core set categories for the HSCT patient are found to be relevant. Nurses explained that the reasons for different levels of relevancy included (i) the stage of disease, (ii) expectations of the patient, and (iii) impact of the disease. “*Yes, all of these ICF categories are relevant, but not in every moment of the disease” (V7 regular nurse)*. Another nurse stated: “*Preparing meals (d630), is not relevant during the admission period, that's only applicable at home” (V1 ICF nurse)*.

### Practicality of the ICF Core Set

From the qualitative analysis, nursing anamnesis and nursing consultations are found to be feasible. The new ICF-related anamnesis was experienced as a good start of the admission. Although it is time-consuming, the effort is worthwhile. “*The time and energy you spent on the anamnesis, pays off during the admission […] it causes less questions and uncertainties by patients later on” (V10 regular nurse)*. The nursing consultations are experienced as thorough conversations and result in valuable information. Opinions of nurses regarding the frequency of nursing consultations depend on changes in condition or functioning of patients. Most nurses mentioned the optimal average frequency of nursing consultations being two or three times a week.

Some difficulties were found related to the use of the mHealth application, especially regarding loading speed (related to the Internet connection) and clarity of the information regarding admitted and discharged patients. “*The overview is not very clear, you can't easily see who and when information is reported” (V9 ICF nurse)*.

### Demand for the ICF Core Set

In the qualitative section of the study, the demand for using the preliminary ICF core set is analyzed from the perspective of patients (articulated by nurses) and the perspective of nurses. From the perspective of patients, using the ICF core set allows (i) patients to receive more attention, (ii) have their needs discussed more frequently, and (iii) give nurses the ability to act on these needs. “*The ICF nurse is able to sit down and take some time for a patient. Patients experience this like: “They have time for me, they listen to me.” “They can try to find a solution for the patients' needs” (V3 regular nurse)*. The nursing consultation creates more awareness about what is important to the patient. This supports self-management and “patient-centered care.” “*The ICF can help the patient to structure their daily lives. What needs to be done and what is required? What can the patient do by himself and where does he need help?” (V5, ICF nurse)*.

From the perspective of nurses, demand for using the ICF core set can be found in improved communication between nurses, physicians, and other disciplines. “*You are able to support your professional input, patient needs are shown more clear” (V10, regular nurse)*. A nurse stated: “*With the ICF framework we can provide relevant information related to patients' daily life in professional terminology. This has an impact on the communication with physicians” (V1 ICF nurse)*. This is also reflected in multidisciplinary meetings. “*The nurse is presenting the patients' information in a clear structure, so that everything is discussed” (V6 ICF nurse)*.

### Acceptability of the ICF Core Set

The acceptability of the ICF core set was discussed in two aspects of healthcare: healthcare provision and the relationship between nurse and patient. Using the information from the nursing consultation, nurses can anticipate potential problems earlier on, consult other disciplines, and determine appropriate healthcare provision. “*With one patient, for example, the issue of continuing his treatment was clarified by the nursing consultations to both nurses and physicians. As a result, the patient was discharged to go home and die” (V4 regular nurse)*. The regular nurses have experienced the ICF project as a valuable addition to regular care of patients. “*I see it as an addition […] it creates a better picture of the patient” (V4 regular nurse)*.

The ICF project adds value to the relationship between patients and nurses. The availability of the ICF nurse in nursing consultation results in continuity of the patient care process. “*Continuity […] that you really know the patient from admission until discharge” (V8 ICF nurse)*. As a result, a trust relationship between a nurse and the patient develops with more in-depth conversations related to functioning. “*You need to create a trust relationship. The more you talk to them, the more they tell you” (V9 ICF nurse)*.

### Implementation of the ICF Core Set

The implementation of the preliminary ICF core set was confronted with facilitators and barriers. There are two facilitators of the implementation indicated by ICF nurses and regular nurses. First, the ICF nurses stated that the close contact between them and the researcher HS was helpful in introducing the concept of functioning and reporting functioning correctly in the standard terminology of the ICF. Second, the regular nurses indicated that the availability of the ICF nurses was complementary to the regular care. The ICF nurses reduced the workload. “*I liked it, it eases the workload” (V3 regular nurse)*.

The most significant barrier was the vulnerability of the organization. “*Illness of the ICF nurses or holidays were a disadvantage for the project because it disturbed the continuity. That's a pity” (V5 ICF nurse)*. Regular care related to HSCT patients is highly biomedical-focused. The limited time of nurses gives biomedical care more priority at the expense of the ICF project and biopsychosocial care.

To enhance the performance of the ICF project, the nurses stated that expanding the amount of ICF nurses in the team is necessary. They explained that time and training is required by using the ICF core set. “*There need to be nurses capable to perform ICF consultations” (V3 regular nurse)*. The most relevant requirement for the project, however, according to the ICF nurses and regular nurses, is a commitment from management. “*Both management support and staffing is required. With their support it will become a success” (V8 ICF nurse)*.

## Discussion

The use of the preliminary ICF core set for HSCT patients provides information about functioning of patients during the hospitalization at the Department of Hematology. The ICF categories structure the communication among the disciplines and facilitate the nurses representing the needs of the patient. Additionally, using the ICF categories in nursing consultations makes patients aware of important aspects of their daily lives. This information is significant for multidisciplinary meetings and may impact the decision-making related to care and treatment plans.

This study provides meaningful insight into the application of the ICF core set in clinical practice. Following the recommendations of previous studies (34, 35), the current study focuses on how to use the ICF core set in clinical practice. Important facilitators, such as efficiency, managerial support, and coaching, were obtained. These facilitators are found in previous studies as well (35, 36). Another benefit of using the ICF core set is regarding multidisciplinary communication. Mainly because the structure of the ICF ensures the evaluation of all aspects of the functioning of patients without a primary focus on disease and disability, as found in other studies (37, 38). The finding that the relevance of ICF categories is related to the stage of the disease is comparable with the study of Scheuringer et al. (39). They found unique aspects of functioning in the post-acute or long-term context. The ICF components Activities and Participation, regarding the relationship, employment, and recreation categories, are used more frequently in the long-term context (39). However, the current study demonstrates that, near to the expected dominance of the most frequently used ICF categories classified in the Body Functions characterizing the acute stage of the disease, also the relationship and employment categories are present in the top 30% most used categories (Table 4). Furthermore, the results of this study show that the frequency of these categories increases with longer hospitalization. This can be explained by the differences in research methods. Scheuringer et al. (39) used a survey to determine the ICF categories by opinions of experts. The current study tested the use of the ICF categories in actual clinical practice. Testing the ICF core set revealed that the top 30% most used ICF categories, except for medication (e110), were all included in the preliminary ICF core set for HSCT patients. The study conducted previously to develop the preliminary ICF core set for HSCT patients did not determine the category medication (e110) (25). This can be explained by the content analysis that was performed at the time based on the daily patient reports, whereas medication (e110) was reported on a different form. Studies in medication management related to HSCT patients demonstrated that adherence is an issue due to the complex medical regimes (40, 41). Family relationships (d760) were the most frequently and highly positively associated ICF category. Due to the qualitative-dominant study design, apart from the associations, no further quantitative analyses are performed on the qualifiers. Related to the literature demonstrating the limited psychometric properties of ICF categories (42), a well-designed quantitative study is required to perform extensive analyses on the qualifiers.

Certain aspects must be considered to interpret the results of the current study. First, due to limited time and practical reasons, such as training and coaching, the study sample included only nurses from the Department of Hematology from one University. Given these specific circumstances, the results are not yet generalizable. Nevertheless, to gain a better understanding of using the ICF core set in clinical practice, a scope for one team was recommended (43). Moreover, it is argued that a “bottom-up” approach will be helpful for the nurses to own the process and results in a successful implementation (44).

Second, the results of this study were collected with semi-structured interviews in an attempt to collect expert opinions of nurses. Consequently, some of the results, especially those involving the patient, were indirect. However, the use of expert opinions is often useful in the early phase of applying a model in clinical practice (28). Another limitation was the mHealth application (30). Disturbances related to Internet connection as well as getting a clear overview of patients hampered an easy registration of functioning of patients. Nevertheless, the application provided a reliable way to report the data related to the used ICF categories and to extract the data for scientific research. Intercoder agreement about items and topics by the researcher HS and the researcher JB enhanced the reliability of the qualitative analysis. Finally, the involvement of an ICF expert (the researcher HS) ensured the content validity and helped the nurses learn the fundamentals and report the ICF categories correctly (35).

The results of the study may bring implications for the practice of nursing. In the current study, a structured terminology usable for communication among disciplines was in demand in the nursing field. The ICF provides the structure and the terminology. This structured terminology allows nurses to provide relevant information regarding the functioning of patients. In addition, the terminology will be recognized by physicians and other disciplines and will probably result in better communication between nurses, physicians, and other relevant health professionals.

## Conclusion

From the perspective of nurses, all ICF categories in the preliminary ICF core set for HSCT patients are determined to be relevant and useful in gaining information regarding functioning. Medication (e110) was the only missing category in the core set and should be considered for inclusion. Family relationships (d760) was the most frequently and highly positively associated ICF category with a very large effect. Information related to the functioning of patients provides the nurses with better insight into the situation of patients. The ICF core set can also help patients recognize what is important in their daily lives. Applying the ICF core set with the anamnesis and the nursing consultations contributed to these results. However, as mentioned in literature and demonstrated in this study, training and a well-working tool are requested to perform the consultations and register information of patients in the right way. Further research is needed to look at the perspective of other professionals and HSCT patients themselves.

## Data Availability Statement

The raw data supporting the conclusions of this article will be made available by the authors, without undue reservation.

## Ethics Statement

This study was conducted according to the principles of the World Medical Association (WMA) Declaration of Helsinki. The Medical Ethics Review Board of the University Medical Center Groningen confirmed that the Dutch Medical Research Involving Human Subjects Act does not apply to the current study. The nurses who participated in the study signed an informed consent before the interview. The patients signed a general informed consent before hospitalisation. All participants were informed that their participation in this study was voluntary and that they could withdraw from the study at any time. All data was anonymised and stored on a secure USB stick and the secured hard drive of the hospital. Only researchers HS and JB had access to the data.

## Author Contributions

HS, JB, SH, and PR designed the study with input from HO-M and MK. HS, JB, and SH developed and planned the preparation and testing phase. JB recruited participants and collected and analyzed data with supervision of HS. RS supervised the statistics. HS and JB led the writing of the manuscript. HO-M, MK, LO, and PR supervised the execution of the study. All authors contributed to the review, editing, and final approval of the manuscript.

## Conflict of Interest

The authors declare that the research was conducted in the absence of any commercial or financial relationships that could be construed as a potential conflict of interest.

## Publisher's Note

All claims expressed in this article are solely those of the authors and do not necessarily represent those of their affiliated organizations, or those of the publisher, the editors and the reviewers. Any product that may be evaluated in this article, or claim that may be made by its manufacturer, is not guaranteed or endorsed by the publisher.
